# The red wine component ellagic acid induces autophagy and exhibits anti‐lung cancer activity in vitro and in vivo

**DOI:** 10.1111/jcmm.13899

**Published:** 2018-10-24

**Authors:** Jing Duan, Ji‐Cheng Zhan, Gui‐Zhen Wang, Xin‐Chun Zhao, Wei‐Dong Huang, Guang‐Biao Zhou

**Affiliations:** ^1^ Beijing Advanced Innovation Center for Food Nutrition and Human Heath College of Food Science and Nutritional Engineering China Agricultural University Beijing China; ^2^ State Key Laboratory of Membrane Biology Institute of Zoology Chinese Academy of Sciences Beijing China

**Keywords:** autophagy, celastrol, CIP2A, ellagic acid, lung cancer

## Abstract

Red wine consists of a large amount of compounds such as resveratrol, which exhibits chemopreventive and therapeutic effects against several types of cancers by targeting cancer driver molecules. In this study, we tested the anti‐lung cancer activity of 11 red wine components and reported that a natural polyphenol compound ellagic acid (EA) inhibited lung cancer cell proliferation at an efficacy approximately equal to that of resveratrol. EA markedly increased the expression of the autophagosomal marker LC3‐II as well as inactivation of the mechanistic target of rapamycin signalling pathway. EA elevated autophagy‐associated cell death by down‐regulating the expression of cancerous inhibitor of protein phosphatase 2A (CIP2A), and CIP2A overexpression attenuated EA‐induced autophagy of lung cancer cells. Treating tumour‐bearing mice with EA resulted in significant inhibition of tumour growth with suppression of CIP2A levels and increased autophagy. In addition, EA potentiated the inhibitory effects of the natural compound celastrol on lung cancer cells in vitro and in vivo by enhancing autophagy and down‐regulating CIP2A. These findings indicate that EA may be a promising chemotherapeutic agent for lung cancer, and that the combination of EA and celastrol may have applicability for the treatment of this disease.

## 1 INTRODUCTION

Lung cancer is a leading cause of cancer‐related mortality in developed and developing countries, which accounts for 1.59 million deaths worldwide each year.^1^ Current drugs (excluding surgery) for this disease include chemotherapy, radiation, targeted therapy, and immunotherapy. However, the 5‐year overall survival rate of lung cancer of all stages combined is no more than 18%.^2^ Novel therapies, for example, novel immunotherapy, cancer hallmarks‐targeting or autophagy‐regulating approaches,^3^ should be developed to combat this neoplasm.

Red wine component resveratrol is a small polyphenol which confers strong protection against metabolic, cardiovascular, and neurodegeneration diseases.^4^ Resveratrol exhibits chemopreventive^5^ and therapeutic effects against several cancers^6^ by targeting some molecules that play important roles in tumourigenesis.^7^ Indeed, other red wine compounds also have anti‐cancer activity.^8^ Ellagic acid (EA), a natural polyphenol compound found in grapes, strawberries, and nuts,^9^ has higher concentration in red wine than resveratrol and reaches 15 mg/L in some Spain red wine.^10^ Recent studies show that EA has antioxidant and preventive effects in several types of cancers.^11^, ^12^, ^13^, ^14^ EA exhibits in vitro and in vivo anticancer activities by arresting the cell cycle, inhibiting tumour cell proliferation, inducing cell apoptosis, ameliorating inflammation, and inhibiting angiogenesis.^15^, ^16^, ^17^, ^18^ EA suppresses Akt, Shh, and Notch pathways^19^, ^20^ and down‐regulates the levels of 17β‐estradiol‐metabolizing enzymes.^21^ However, the effects and underlying molecular mechanisms of EA in lung cancer remain to be investigated.

Cancerous inhibitor of protein phosphatase 2A (CIP2A) or KIAA1524/p90^22^, ^23^ is an oncoprotein which promotes tumourigenesis through facilitation of the biological functions of cancer drivers (c‐Myc, Akt, etc.) and inhibition of PP2A activity.^24^ CIP2A controls autophagy and cell growth through mTORC1 activation,^25^ synergizes with RAS,^26^, ^27^ and crosstalks with Wnt/β‐catenin signal pathway^28^ to promote cell proliferation and cancer progression.^23^, ^29^ It also mediates interleukin 10 (IL‐10)‐induced tumour aggressiveness.^30^ CIP2A is overexpressed at a high frequency in most types of human cancers, inversely correlated with disease outcome of the patients,^24^ and is associated with doxorubicin resistance.^31^ CIP2A is recognized as a “druggable” target, and compounds including celastrol,^32^ ethoxysanguinarine,^33^ and rabdocoetsin B^29^ inhibit CIP2A and induce apoptosis of lung cancer cells. CIP2A is targeted by some kinase inhibitors^34^, ^35^, ^36^ and proteasome inhibitor^37^ to exert anti‐cancer activity. Therefore, identifying novel CIP2A inhibitors may provide optimal drug candidates for clinical testing.

In this study, we tested the effects of 11 red wine polyphenolic compounds on lung cancer cells and found that EA had the strongest anticancer effects that significantly suppressed tumour growth in vivo. Moreover, the mechanistic studies showed that EA induces autophagy by targeting CIP2A in lung cancer cells.

## 2 MATERIALS AND METHODS

### 2.1 Compounds and antibodies

Resveratrol, ferulic acid, epicatechin, EA, rutin, chlorogenic acid, coumalic acid, vanillic acid, syringic acid, morin, phloridzin, bafilomycin A1, and 3‐Methyladenine (3‐MA) were purchased from Sigma (St Louis, MO, USA). Celastrol was purchased from Pie & Pie Technologies (Shenzhen, Guangdong, China). For experimental use, EA was dissolved in 1 M NaOH (Xilong Chemical, Guangdong, China), and all the other compounds were dissolved in dimethyl sulphoxide (DMSO) (Amresco, Solon, OH, USA). The final concentration of NaOH and DMSO was ≤0.1% (v/v) and did not contribute to toxicity.

Antibodies used in this study included anti‐LC3, anti‐p62/SQSTM1, and anti‐ATG5 (Sigma), anti‐Akt, anti‐phosphor‐Akt (Ser473), antimechanistic target of rapamycin (mTOR), anti‐phosphor‐mTOR (Ser2448), anti‐P70S6K, and anti‐phosphor‐P70S6K (Thr412/389; Cell Signaling Technology, Beverly, MA, USA), anti‐CIP2A, c‐Myc, and anti‐TIM23 (Santa Cruz Biotechnology, Dallas, TX, USA) antibodies. For Western blot, antibodies used were diluted 1:500 to 1:2000. For immunohistochemistry assay, antibodies were diluted 1:200 or 1:1000.

### 2.2 Cell culture and transfection

The lung adenocarcinoma cell lines HOP62 and H1975 (harbouring L858R/T790M EGFR mutation) were purchased from Cell Bank of Chinese Academy of Sciences (Shanghai, China) and American Type Culture Collection (Manassas, VA, USA) respectively. The cells were cultured in Dulbecco's Modified Eagle's Medium or RPMI 1640 (Invitrogen, Carlsbad, CA, USA) supplemented with 10% foetal bovine serum (Gibco BRL, Grand Island, NY, USA) and 1% penicillin‐streptomycin. Cells were cultured at 37°C in an atmosphere of 95% air and 5% CO_2_ under humidified condition.

The pcDNA3.1‐CIP2A construct (provided by Professor Jukka Westermarck at University of Turku, Finland) and siRNA for ATG5 (siATG5; Dharmacon, Lafayette, CO, USA) were transfected into the cells using Lipofectamine 3000 (Invitrogen), and 48 hours later, the cells were treated with or without 50 μM EA for additional 24 hours.

### 2.3 Cell proliferation, cell viability, and clonogenic assays

The cancer cells (5000 cells per well) were seeded in each well of 96‐well microplates. Eighteen hours after seeding, new medium containing different concentrations of target compounds or solvent control was added. MTT (3‐(4,5)‐dimethylthiahiazo (‐z‐y1)‐3,5‐di‐phenytetrazoliumromide; Amresco, Solon, OH, USA) assay was performed to assess the effects of compounds on cancer cell proliferation. The dose‐effect curves of single or combined drug treatment were analysed by the median‐effect method of Chou and Talalay using the Calcusyn Software (Biosoft, Cambridge, UK), and the combination indexes (CI) less than, equal to, and greater than 1 indicated synergistic, addictive, and antagonistic effects, respectively. Cell viability was determined by trypan blue dye exclusion assay. For cell cycle analysis, the cells were treated with EA at different concentrations for 24 hours. The cells were centrifuged and washed with phosphate‐buffered saline (PBS), followed by incubation with RNase and propidium iodide (PI) (Sigma). Cell cycle distribution was analysed by flow cytometry (BD FACS Vantage Diva, Franklin Lakes, NJ, USA). Cell apoptosis was measured using Annexin V/PI Apoptosis Detection kit (BD Biosciences, San Jose, CA, USA) according to manufacturer's instruction. For foci formation assay, HOP62 or H1975 cells were treated with or without EA and seeded in triplicate into six‐well culture plates (300 cells per well). After 8 days of culturing, the colonies were fixed in methanol and stained with 0.005% crystal violet (Sigma). Clones containing more than 50 cells were counted.

### 2.4 Immunofluorescence analysis

Cells (1.5 × 10^5^ per well) were seeded on coverslips with 1% gelatin in six‐well culture plates and grown to 60% confluence. After treatment, cells were washed three times with PBS and fixed in 4% paraformaldehyde for 15 minutes at room temperature. Fixed cells were permeabilized in 0.3% Triton X‐100 for 20 minutes. The cells were blocked by incubating with 5% bovine serum in PBS with Tween 20 (PBS‐T) for 30 minutes, incubated with an anti‐LC3 antibody overnight at 4°C, washed again with PBS‐T, and stained with a fluorescent labelled secondary antibody for 2 hours at room temperature in a dark humid chamber. The cells were mounted and visualized under a confocal microscope (A1R‐STORM; Nikon, Tokyo, Japan).

### 2.5 Western blot

Cells were lysed in RIPA buffer (50 mM Tris‐HCl [pH 7.4], 150 mM NaCl, 0.1% SDS, 1% sodium deoxycholate, 1% TritonX‐100, 1 mM EDTA) supplemented with protease inhibitors. Equivalent protein quantities (20 μg) were separated by 10%‐15% sodium dodecyl sulphate polyacrylamide gel electrophoresis and electrotransferred onto a nitrocellulose membrane. Then, the membrane was blocked in 5% nonfat milk in Tris‐buffered saline and incubated with the indicated primary antibodies, followed by the appropriate secondary antibodies. Proteins were detected using the Luminescent Image Analyser LSA 4000 (GE, Fairfield, CO, USA). Western blot data were quantified by densitometry analysis and the relative expression values to Actin were listed under respective bands.

### 2.6 Quantitative PCR

Total RNAs were extracted using the Trizol reagent (Invitrogen) and the phenol‐chloroform extraction method according to the manufacturer's instructions. To detect the mRNA expression of related genes, quantitative PCR (qPCR) was conducted with SYBRTM Green Real time PCR Master Mix (Takara Biotechnology, Dalian, China). The following primers were used in this study: *CIP2A*, forward 5′‐GCCACACTGATTCGGTGTTTT‐3′, reverse 5′‐TGCCGACAAAGATTTGCCAA TA‐3′; *GAPDH*, forward 5′‐GAGTCAACGGATTTGGTCGT‐3′, reverse 5′‐GACAA GCTTCCCGTTCTCAG‐3′.

### 2.7 Enzyme‐linked immunosorbent assay

Mouse blood samples were obtained before cervical dislocation. Serum concentration of creatine (Cr), aspartate transaminase (ALT), and alanine transaminase (AST) was determined using a commercially available enzyme‐linked immunosorbent assay kit (Shanghai GenePharma, Shanghai, China). The absorbance was read at 450 nm using an automated Microplate Reader (Bio‐Tek, Winooski, VT, USA).

### 2.8 Animals

The animal studies were approved by the Institutional Review Board of the Institute of Zoology, Chinese Academy of Sciences. Female BALB/C nude mice (5‐6 weeks old) were purchased from Vital River Laboratory (Beijing, China) and treated in accordance with the guidelines of the Animal Use and Care Committee of the Institute of Zoology, Chinese Academy of Sciences. HOP62 cells (1 × 10^6^) were injected subcutaneously into the right rear flank of each mouse. After 4 days, the mice were randomized into five groups and treated with EA (0, 40, 80 mg/kg) via oral gavage or celastrol (1 mg/kg) via intraperitoneal injection in corn oil once every 2 days for 22 days. Tumour size was measured every alternate day with an electronic caliper and calculated with the following formula: volume (mm^3^) = 1/2 (width)^2^ × length. After 22 days of treatment, the mice were always killed by cervical dislocation. Tumour tissues were excised, homogenized, and lysed for Western blot analysis.

### 2.9 Statistical analysis

All experiments were repeated at least three times and the data are presented as the mean ± SD. Differences between data groups were evaluated for significance using the Student's *t* test of unpaired data (two‐tailed). For animal studies, the data are presented as the mean ± SEM. The tumour volume was analysed with one‐way ANOVA and then Tukey's test after ANOVA to identify the pairs with significant differences using the software SPSS 16.0 for Windows (Chicago, IL, USA). *P* < 0.05 was considered statistically significant.

## 3 RESULTS

### 3.1 Antiproliferative effects of red wine polyphenolic compounds

**Figure 1:**
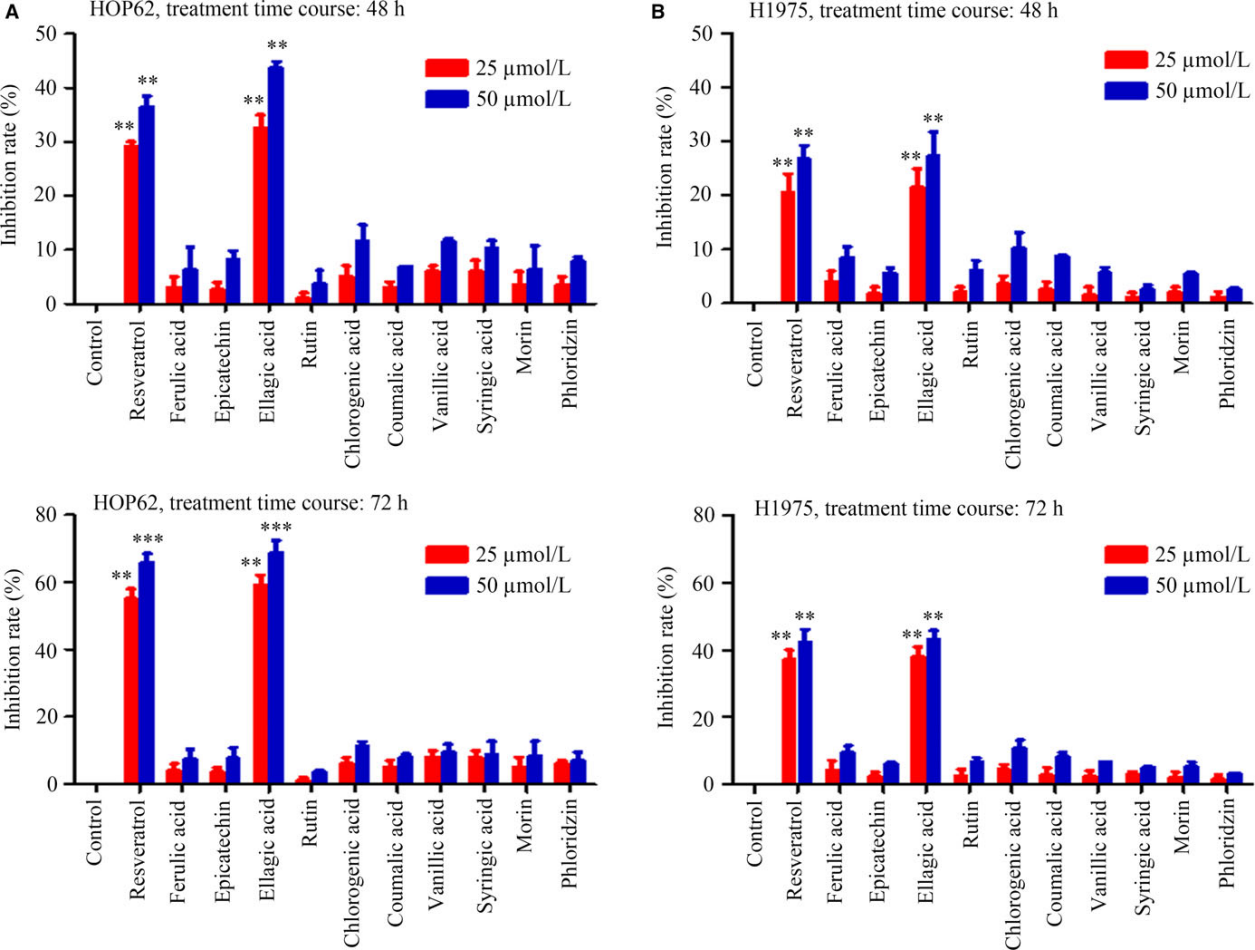
Antiproliferative activities of 11 red wine compounds in lung cancer cell lines. (A) HOP62 and (B) H1975 cells were treated with indicated compounds (25 and 50 μM) for 48 and 72 hours, and cell viability was detected by the MTT assay. Data are presented as the mean ± SD. **P < 0.01; ***P < 0.001

To identify red wine compounds that have anticancer activities, MTT assay was performed in HOP62 (Figure 1A) and H1975 (Figure 1B) cells treated with 11 polyphenolic compounds (resveratrol, ferulic acid, epicatechin, EA, rutin, chlorogenic acid, coumalic acid, vanillic acid, syringic acid, morin, and phloridzin). Nine of the compounds only exhibited low proliferation inhibition rate on the cells (<15%). Interestingly, EA and resveratrol showed significant inhibition rate in the cells (Figure 1).

### 3.2 Inhibitory effects of EA on lung cancer cells

**Figure 2:**
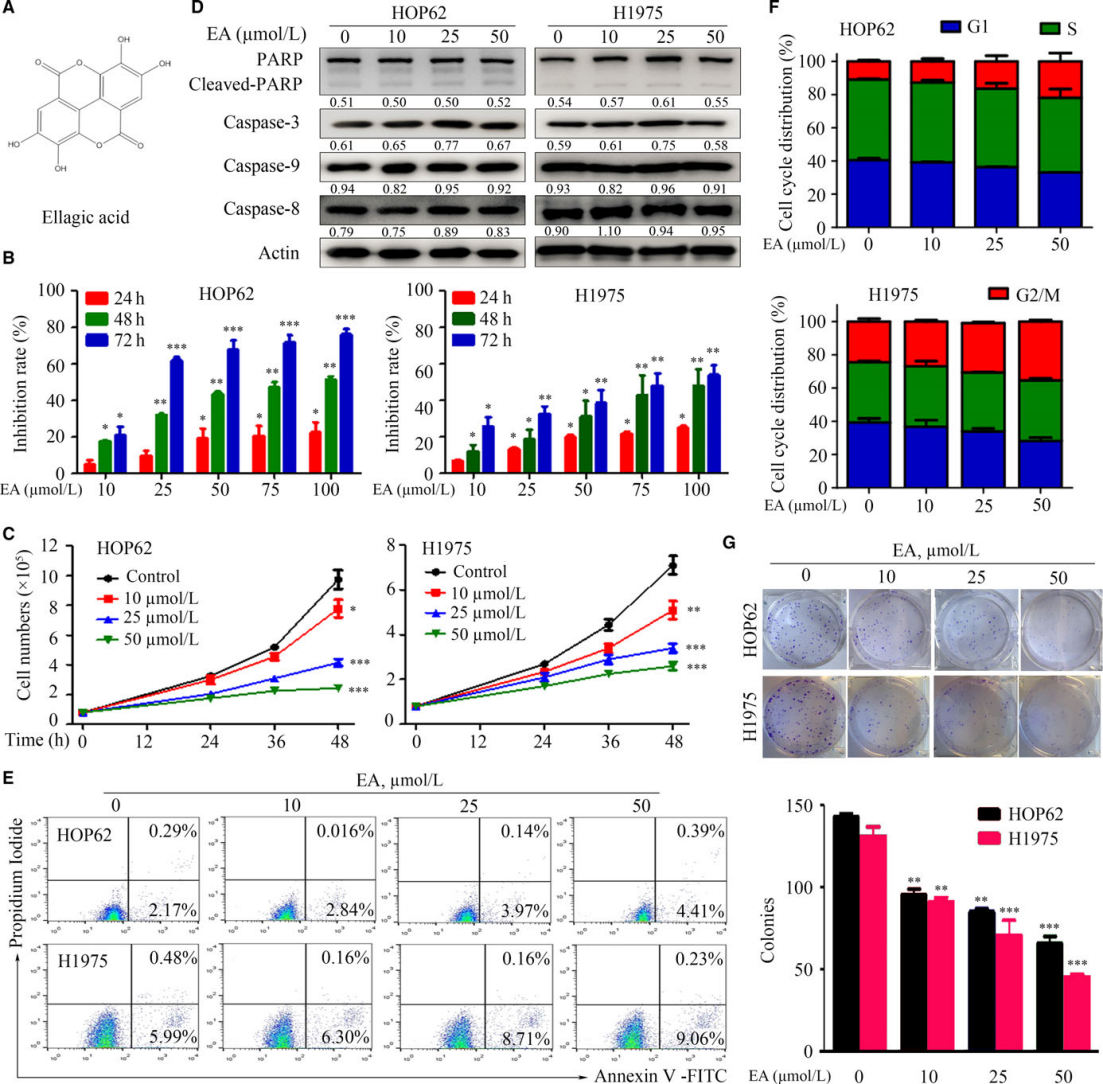
EA exhibits cytotoxicity and decreases viability of lung cancer cell lines. (A) Structures of EA. (B) HOP62 and H1975 cells were treated with EA (10‐100 μM) for 24, 48, and 72 hours, and cell viability was detected by the MTT assay. (C) HOP62 and H1975 cells were treated with different concentrations of EA for the indicated time‐points and assessed by trypan blue exclusion analysis. (D) Western blot assays of EA‐treated cell lysates with indicated antibodies. Numbers under the bands are the relative expression values to Actin determined by densitometry analysis. (E) EA‐treated cells were analysed by Annexin V/PI and flow cytometry. (F) Cell cycle distribution of EA‐treated cells. (G) Colony formation assay for HOP62 and H1975 cells treated with or without EA (10, 25, 50 μM). Data are presented as the mean ± SD. *P < 0.05; **P < 0.01; ***P < 0.001

To evaluate the effects of EA (Figure 2A) in lung cancer cells, MTT and trypan blue dye exclusion assays were performed in HOP62 and H1975 cells treated with different concentrations of EA for the indicated time‐points. The results showed that EA inhibited cancer cell proliferation and growth in a time‐ and dose‐dependent manner (Figure 2B and C). EA did not induce significant apoptosis of lung cancer cells, confirmed by lack of PARP cleavage, Casp‐3 activation (Figure 2D), and Annexin V‐positive cells (Figure 2E). EA only slightly arrested cell cycle at G2/M phase (Figure 2F). The colony formation assay showed that the addition of EA to HOP62 and H1975 cells significantly decreased the colony formation activity of the cells (Figure 2G).

### 3.3 EA induces autophagy in lung cancer cells

**Figure 3:**
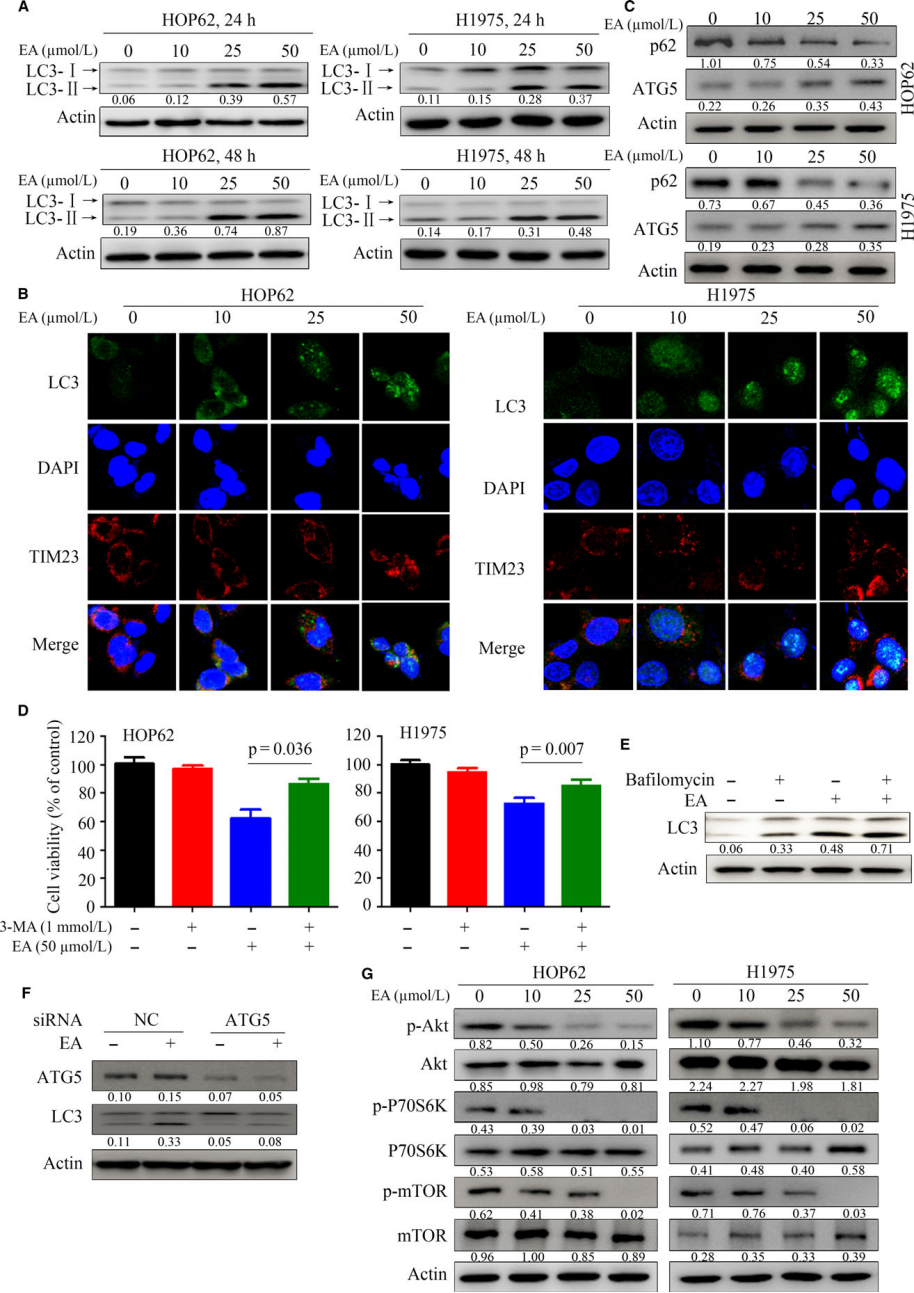
EA induces autophagy in lung cancer cells. (A) HOP62 and H1975 cells were treated with EA for 24 hours and LC3‐I and LC3‐II levels were analysed by Western blot. Numbers under the bands (LC3‐II for LC3) are the relative expression values to Actin determined by densitometry analysis. (B) HOP62 cells were treated with EA at different concentrations for 24 hours and assessed by immunofluorescence analysis using an anti‐LC3 antibody. (C) Western blot was performed to detect the expression of indicated proteins in HOP62 and H1975 cells treated with EA for 24 hours. (D) The cells were treated with EA and/or 3‐MA, and the viable cells were tested by MTT assay. (E) Western blot assays of lysates of HOP62 cells upon EA/bafilomycin A1 treatment. (F) The HOP62 cells were transfected with siNC or siATG5 and 24 hours later treated with EA for additional 24 hours. The cells were lysed and the lysates were subjected to Western blot using indicated antibodies. (G) Lysates of EA‐treated cells were subjected to Western blot with indicated antibodies. Numbers under the bands are the relative expression values to Actin determined by densitometry analysis

To test whether EA could induce autophagy in lung cancer cells, HOP62 and H1975 cells were treated with EA for 24 and 48 hours. The cells were then lysed and the lysates were subjected to Western blot to assess the changes in the cytosolic form (LC3‐I) and lipidated form (LC3‐II) of the LC3 autophagic activity marker.^38^ Notably, EA induced accumulation of LC3‐II in HOP62 and H1975 cells in a dose‐dependent manner (Figure 3A). To confirm the presence of LC3‐II accumulation in the autophagic process, the formation of green fluorescent protein (GFP)‐tagged LC3‐positive autophagosome was examined. We found that EA induced a significant increase in GFP‐LC3 puncta in the nucleus and mitochondria (Figure 3B). In addition, EA treatment reduced protein levels of p62/SQSTM1, a well‐established receptor for autophagic cargo and substrate for autophagic degradation, and increased the levels of ATG5, an essential molecule for the induction of autophagy (Figure 3C). The results of MTT assays showed that the cell viability inhibition of EA was attenuated by autophagy inhibitor 3‐MA in both HOP62 and H1975 cells (Figure 3D). Moreover, lysosomal acidification inhibitor Bafilomycin A1 promoted LC3‐II accumulation in EA‐treated lung cancer cells (Figure 3E). In addition, we showed that knockdown of ATG5 by siRNA blocked the formation of LC3‐II in EA‐treated HOP62 cells (Figure 3F). These results indicated that EA induced autophagy and enhancement of the autophagic flux.

The mTOR is an Akt signalling protein and a critical regulator of cellular metabolism, growth, and proliferation, with p70 ribosomal protein S6 kinase (p70S6K) functioning as an important effector.^39^ Recent studies have reported that the Akt‐mTOR pathway plays a key role in autophagy process.^40^ We showed that EA down‐regulated phosphorylated Akt (p‐Akt) and inhibited mTOR phosphorylation as well as p70S6K in both HOP62 and H1975 cells (Figure 3G).

### 3.4 EA induces autophagy through down‐regulation of CIP2A

**Figure 4:**
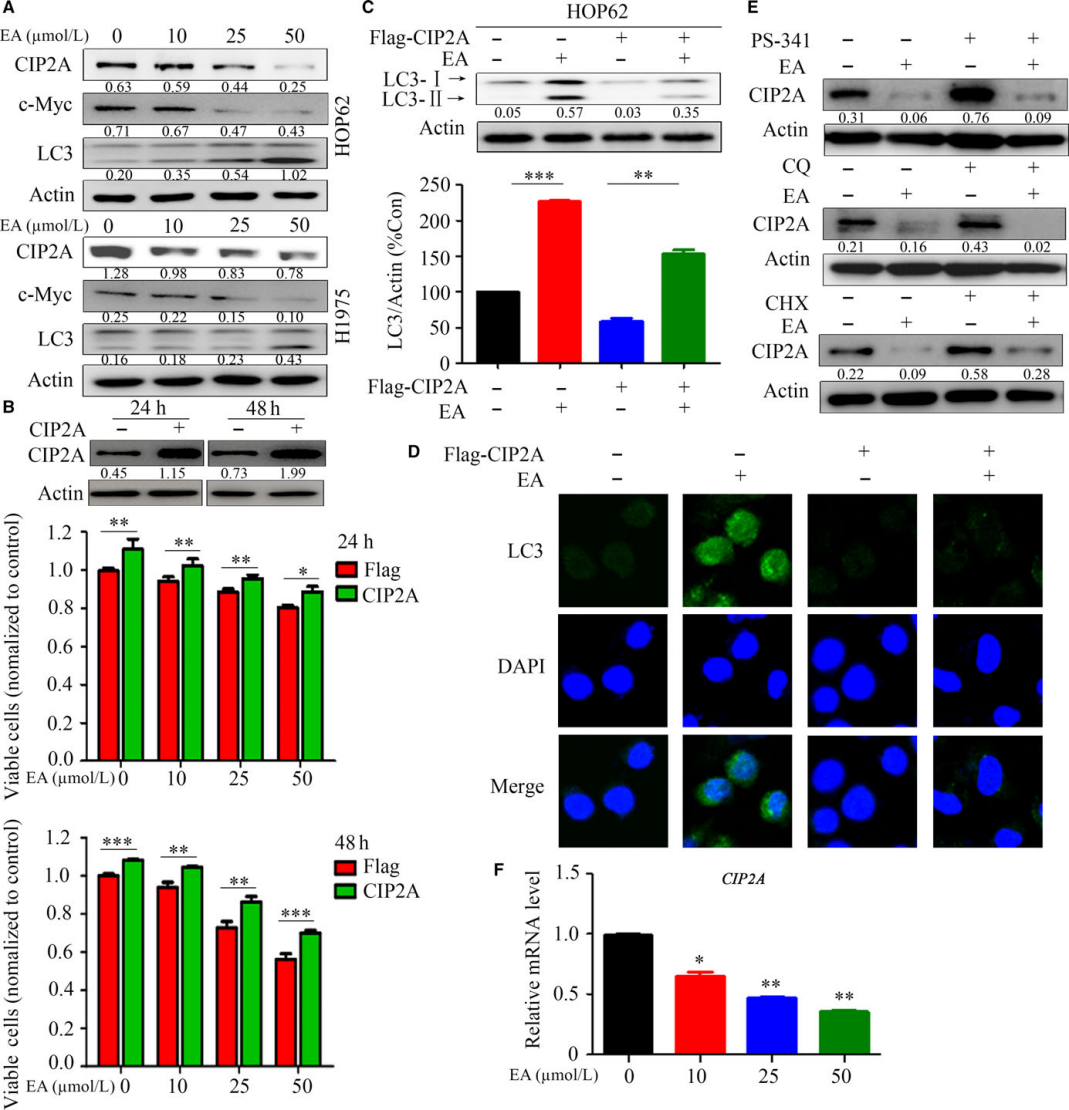
EA treatment leads to CIP2A down‐regulation and overexpression of CIP2A attenuates EA‐induced autophagy. (A) Western blot analysis of CIP2A, c‐Myc, and LC3 in HOP62 and H1975 cells treated with EA. Numbers under the bands (LC3‐II for LC3) are the relative expression values to Actin determined by densitometry analysis. (B) HOP62 cells were transfected with CIP2A and treated with EA for 24 and 48 hours, and viable cells were detected by the MTT assay. The expression of CIP2A was tested by Western blot assay. Numbers under the bands are the relative expression values to Actin determined by densitometry analysis. (C, D) HOP62 cells were transfected with CIP2A and treated with EA for 24 hours; the cells were subjected to Western blot assays (C) and immunofluorescence analysis (D). Data are presented as the mean ± SD;* P*‐values were determined with the *t* test. (E) HOP62 cells were treated indicated compounds for 24 hours, lysed, and the lysates were subjected to Western blot to test the expression of CIP2A. Numbers under the bands (LC3‐II for LC3) are the relative expression values to Actin determined by densitometry analysis. (F) HOP62 cells were treated with EA for 24 hours, harvested, and the expression of *CIP2A* was detected qPCR. *P < 0.05; **P < 0.01; ***P < 0.001

To examine how autophagy is induced by EA, the expression of CIP2A was detected by Western blot. We found that CIP2A in lung cancer cells was dramatically decreased upon EA treatment in a dose‐dependent manner (Figure 4A). In the cells upon EA, the expression of c‐Myc was down‐regulated while LC3‐II was up‐regulated (Figure 4A). To determine the role of CIP2A in EA‐induced autophagy, HOP62 cells were transfected with *CIP2A*‐containing plasmids and treated with or without EA. The MTT analysis showed that overexpression of CIP2A promoted cell growth and attenuated the inhibition of cell proliferation by EA treatment (Figure 4B). CIP2A overexpression markedly decreased LC3‐II protein level in cells treated with EA (Figure 4C). The results were confirmed by immunofluorescence staining with an anti‐LC3 antibody in CIP2A‐expressing cells coincubated with or without EA (Figure 4D). Collectively, these results suggest that EA induced autophagy to inhibit lung cancer cell proliferation by down‐regulating CIP2A.

We examined the mechanism underlying CIP2A down‐regulation in cells treated with EA. The proteasome inhibitor PS341 (100 nM), lysosomal protease inhibitor chloroquine (80 μM), and protein synthesis inhibitor cycloheximide (CHX, 50 μg/mL) were used to treat cells in the absence or presence of EA. The results showed that these inhibitors failed to prevent CIP2A down‐regulation (Figure 4E). Instead, qPCR showed that treatment with EA decreased *CIP2A* mRNA in HOP62 cells, indicating that CIP2A down‐regulation triggered by EA is regulated at the transcriptional level (Figure 4F).

### 3.5 In vivo anti‐lung cancer activity of EA

**Figure 5:**
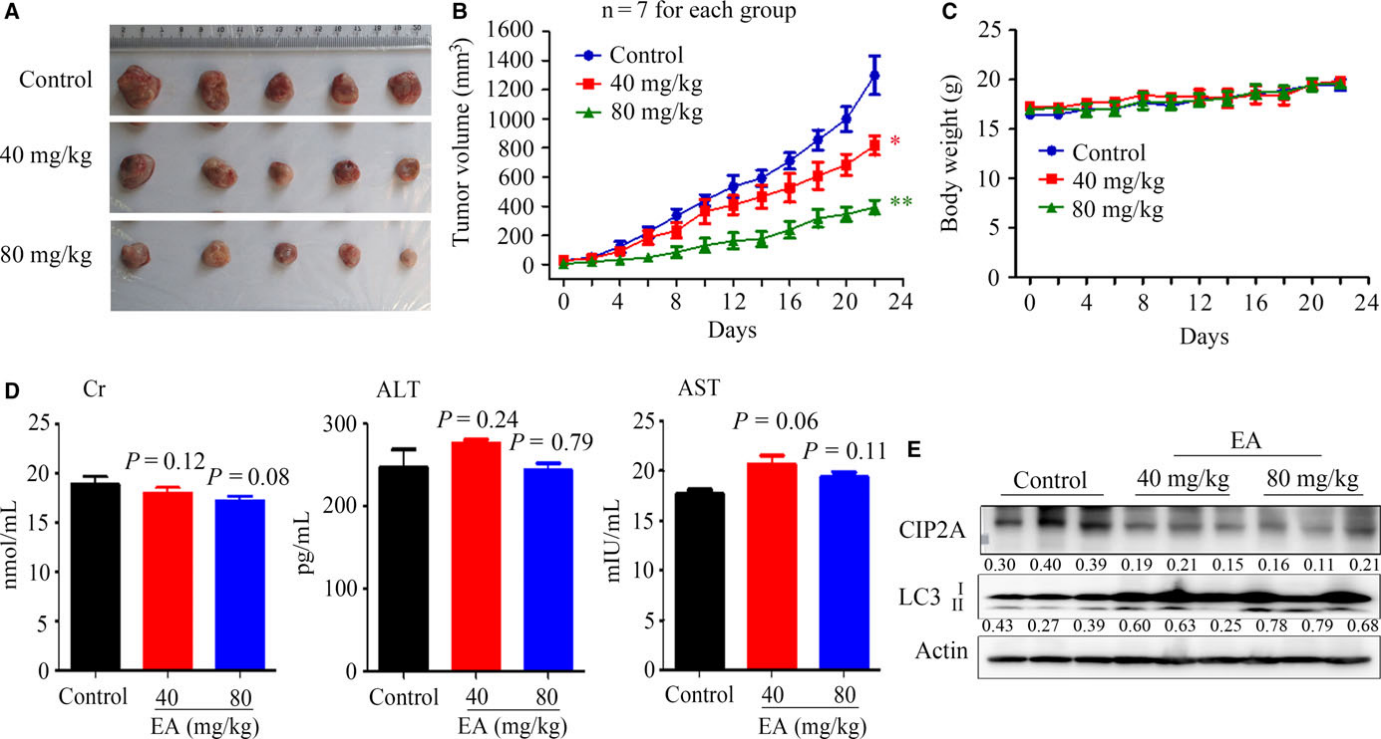
In vivo anti‐lung cancer efficacy of EA. (A) Images of xenograft tumours obtained from the mice (n = 7 for each group). HOP62 cells were inoculated subcutaneously into the right flank of nude mice, which were treated with the indicated concentrations of EA. (B) Efficacy of EA on tumour growth in nude mice injected with HOP62 cells. Data are presented as the mean ± SEM. *P* = 0.016 for one‐way ANOVA;* P*‐values for comparison between control group and each treatment group: *P < 0.05; **P < 0.01. (C) The body weight of mice was monitored every 2 days. (D) The serum levels of Cr, ALT, and AST in mice from each group were detected. (E) Western blot assays using lysates of isolated tumours and indicated antibodies. Numbers under the bands (LC3‐II for LC3) are the relative expression values to Actin determined by densitometry analysis

To evaluate the anti‐lung cancer activity of EA and examine whether CIP2A is important for autophagy induction in vivo, nude mice were injected subcutaneously with HOP62 cells and treated with EA. The results demonstrated that EA significantly suppressed tumour growth, as reflected by a decrease in tumour volume (Figure 5A). The tumours grew more slowly in EA‐treated mice compared to control mice, and tumour size dramatically decreased in a dose‐dependent manner by EA (Figure 5A and B). In addition, EA treatment did not lead to a reduction in body weight (Figure 5C). Mice treated with EA had normal serum concentrations of ALT, Cr, and AST compared to control mice (Figure 5D), indicating that EA treatment did not lead to liver or kidney toxicity. Moreover, Western blot analysis revealed that EA‐treated mice showed a marked decrease in CIP2A levels and an increase in LC3 levels (Figure 5E). Thus, EA treatment induced autophagy and down‐regulation of CIP2A.

### 3.6 EA synergizes with celastrol in inhibiting lung cancer cells

**Figure 6:**
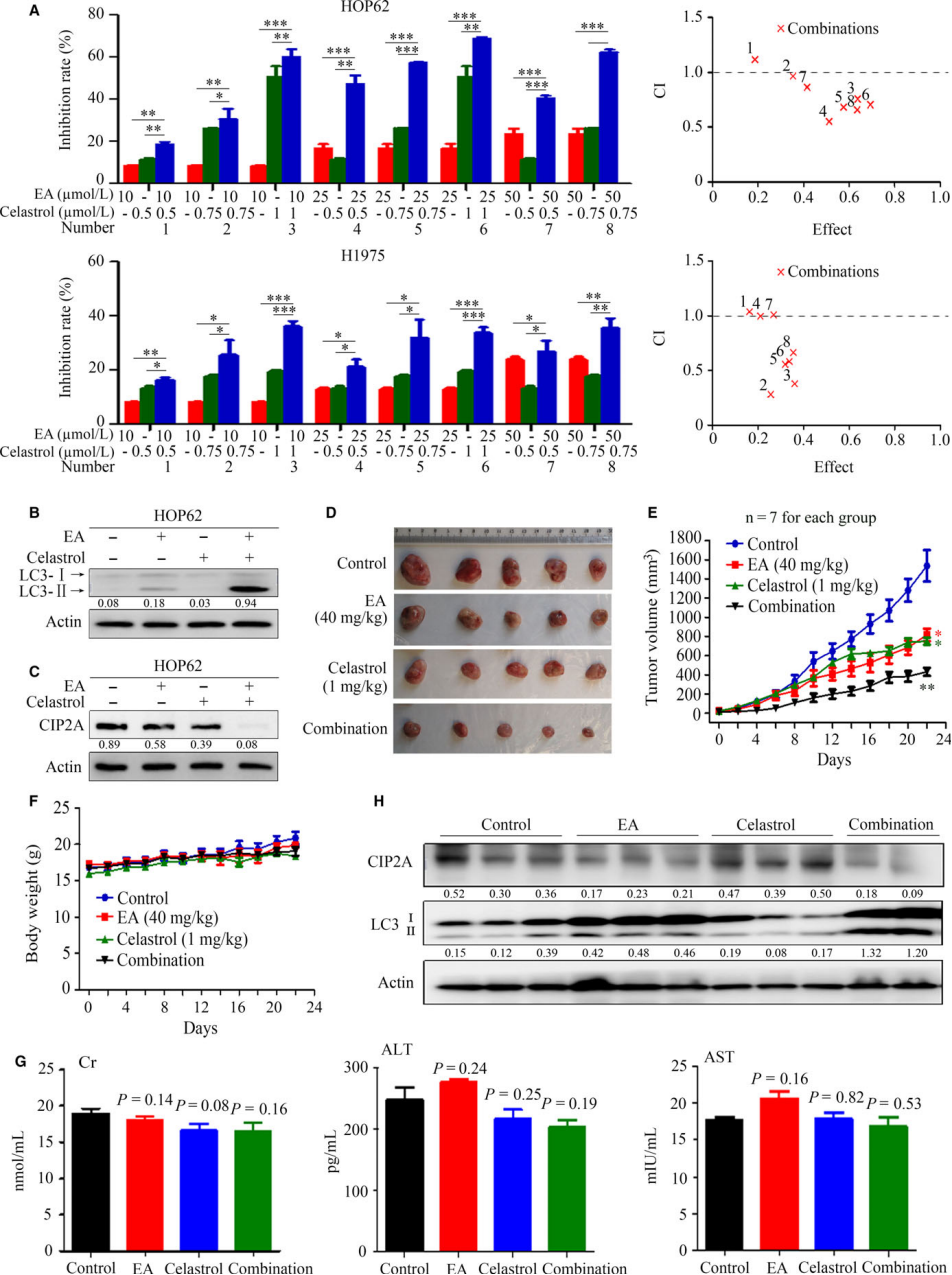
Combined effects of EA and celastrol in mice injected with lung cancer cells. (A) HOP62 and H1975 cells were treated with EA and/or celastrol for 24 hours, and cell proliferation was detected by MTT assay. CI plots were generated by the Chou‐Talay method and Calcusyn software. The numbers 1‐8 correspond to the number labelled representing different treatment combinations. (B, C) HOP62 cells were treated with EA (25 μM) and/or celastrol (0.75 μM) for 24 hours, and LC3 (B) and CIP2A (C) were analysed by Western blot. (D) Images of xenograft tumours obtained from the mice. Nude mice‐bearing HOP62 cells were treated with EA and/or celastrol. (E) Efficacy of the EA and celastrol combination on tumour growth in nude mice injected with HOP62 cells. Tumour volume was measured every 2 days, n = 7 for each group. Data are presented as the mean ± SEM. *P* = 0.032 for one‐way ANOVA;* P*‐values for comparison between control group and each treatment group: *P < 0.05; **P < 0.01. *P*‐values for comparison between combined treatment group and EA alone or celastrol alone group: *P* = 0.04, respectively. (F) The body weight of mice was monitored every 2 days. (G) The serum levels of Cr, ALT, and AST in mice from each group were detected. (H) Tumour samples from mice were harvested, homogenized, and lysed for Western blot using the indicated antibodies. Numbers under the bands (LC3‐II for LC3) are the relative expression values to Actin determined by densitometry analysis

Celastrol is a natural compound isolated from a Traditional Chinese Medicinal herb thunder god vine or *Tripterygium wilfordii* Hook F. which shows potent anti‐lung cancer activity through induction of CIP2A proteasomal degradation.^32^ To examine the combined effects of celastrol and EA, HOP62 and H1975 cells were treated with celastrol and/or EA and evaluated by the MTT assay. The inhibition rates of the compounds on the cells were assessed by Calcusyn Software, and the dose‐effect curves of single or combined drug treatment were analysed by the median‐effect method. The results showed that 10‐50 μM EA significantly enhanced the effects of celastrol (at relatively low concentrations) on lung cancer cells, with CI values less than 1, indicating that the combined effects were synergistic (Figure 6A). To determine whether EA combined with celastrol also induced autophagy, the expression of LC3‐II was analysed in HOP62 cells. Indeed, the combination treatment further enhanced LC3‐II expression compared to treatment with EA (25 μM) or celastrol (0.75 μM) alone in cells (Figure 6B). Consistent with this observation, CIP2A in cells treated with EA and celastrol were significantly reduced compared with control cells (Figure 6C).

We evaluated the therapeutic potential of combined use of EA and celastrol in mice‐bearing HOP62 cells. To this end, nude mice were inoculated with HOP62 cells and subjected to EA (40 mg/kg), celastrol (1 mg/kg), or EA combined with celastrol treatment for 22 days. We found that combined treatment resulted in significant inhibitory effects on tumour growth compared with either monotherapy (Figure 6D and E), without a reduction in body weight (Figure 6F), or increase in serum concentrations of Cr, ALT, and AST (Figure 6G). Moreover, combined use of EA and celastrol up‐regulated LC3‐II and down‐regulated CIP2A compared to each monotherapy (Figure 6H). These data indicate that the combination of EA and celastrol exerts synergistic anti‐lung cancer effects both in vitro and in vivo.

## 4 DISCUSSION

Polyphenols are believed to mediate many of the health benefits of moderate wine consumption. Some of these polyphenols have inhibitory effects and chemoprevention potentials in several types of cancers.^41^ Here, we examined the potential anti‐lung cancer effects of 11 red wine polyphenols and found that EA exhibited almost the same effect as resveratrol in lung cancer (Figure 1). EA was capable of inhibiting lung cancer cell proliferation and inducing autophagy, which was regulated by CIP2A oncoprotein. Previous studies showed that EA did not affect the viability of normal cells.^16^, ^42^ We showed that EA inhibited tumour growth in nude mice‐bearing HOP62 cells without obvious side effects, as determined by the measurements of body weight as well as serum concentrations of Cr, ALT, and AST (Figure 5). As EA monotreatment with a lower dosage only modestly inhibited tumour growth, we hypothesized that combining EA with other known anticancer agents may increase its therapeutic benefits. Among the drugs tested in a screen of human lung cancer cell lines, we found that celastrol at a very low dosage with no apparent toxicity exhibited synergistic effects with EA in inhibiting lung cancer cells and tumour growth (Figure 6). These results indicate that EA might have the potential to be developed as a therapeutic agent in lung cancer.

The role of autophagy in cancer is diverse. Autophagy and autophagy‐related processes promote different effects even in a single tumour, depending on the stage of disease progression, cell type, oncogenic drivers, and intensity of the activating signal.^43^ The tumour inhibition role of autophagy in certain human cancers, including nonsmall cell lung cancer, is increasingly being reported.^44^, ^45^ Therefore, it is important to distinguish between the cytoprotective and cytotoxic effects of autophagy in tumourigenesis. In this study, we reported that EA induced autophagy in lung cancer cells (Figure 3), with decreases in p‐Akt, p‐mTOR, and p‐P70S6K (Figure 3E). CIP2A is an oncoprotein which is critical in controlling autophagy.^25^ We found that CIP2A might mediate EA‐induced autophagy in lung cancer cells because overexpression of CIP2A attenuated EA‐induced autophagy (Figure 4). In accordance with this finding, EA dramatically suppressed tumour growth in a nude mouse model of lung cancer by activating autophagy and down‐regulating CIP2A levels (Figure 5). However, the decrease in CIP2A expression by EA treatment was not mediated by proteasomal degradation because proteasome inhibitor did not inhibit EA‐induced CIP2A down‐regulation (Figure 4). EA down‐regulated *CIP2A* at mRNA level (Figure 4), similar to another natural compound rabdocoetsin B which down‐regulates *CIP2A* at mRNA level.^29^ These results indicate that EA is a CIP2A inhibitor and an autophagy inducer in lung cancer.

Combination therapy has been advocated for more than 2500 years by prescriptions called formulae^46^ in Traditional Chinese Medicine to treat diseases. These formulae usually consist of several types of medicinal herbs to exert synergic efficacies with minimized side effects.^47^ We showed that combination of EA and celastrol at low concentrations resulted in enhanced autophagy and in vivo tumour suppression (Figure 6). EA down‐regulated CIP2A through reducing *CIP2A* mRNA expression level (Figure 4), while celastrol induces proteasomal degradation of CIP2A.^32^ These different mechanisms on CIP2A down‐regulation may explain the synergistic anti‐lung cancers effects of EA in combination with celastrol in vitro and in vivo. Red wine and the medicinal herb *T. wilfordii* Hook F have been used by humans for a long history, thus the combined regimen of EA and celastrol could be tested in clinical study for its anti‐lung cancer efficacy.

## CONFLICT OF INTEREST

No potential conflicts of interest were disclosed.
